# The Role of lncRNAs in the Protective Action of Tamoxifen on the Ovaries of Tumor-Bearing Rats Receiving Cyclophosphamide

**DOI:** 10.3390/ijms252312538

**Published:** 2024-11-22

**Authors:** Sylwia Swigonska, Anna Nynca, Tomasz Molcan, Brian K. Petroff, Renata E. Ciereszko

**Affiliations:** 1 Department of Biochemistry, University of Warmia and Mazury in Olsztyn, Prawochenskiego 5, 10-720 Olsztyn, Poland; 2 Department of Animal Anatomy and Physiology, University of Warmia and Mazury in Olsztyn, 10-719 Olsztyn, Poland; anna.nynca@uwm.edu.pl (A.N.); reniac@uwm.edu.pl (R.E.C.); 3 Molecular Biology Laboratory, Institute of Animal Reproduction and Food Research, Polish Academy of Sciences, Tuwima 10, 10-748 Olsztyn, Poland; t.molcan@pan.olsztyn.pl; 4 Department of Pathobiology and Diagnostic Investigation, Michigan State University, East Lansing, MI 48824-1314, USA; bpetroff@msu.edu

**Keywords:** long noncoding RNA (lncRNA), tamoxifen, cyclophosphamide, ovarian reserve, fertility preservation, cancer treatment

## Abstract

Infertility due to ovarian toxicity is a common side effect of cancer treatment in premenopausal women. Tamoxifen (TAM) is a selective estrogen receptor modulator that prevented radiation- and chemotherapy-induced ovarian failure in preclinical studies. In the current study, we examined the potential regulatory role of long noncoding RNAs (lncRNAs) in the mechanism of action of TAM in the ovaries of tumor-bearing rats receiving cyclophosphamide (CPA) as cancer therapy. We identified 166 lncRNAs, among which 49 were demonstrated to be differentially expressed (DELs) in the ovaries of rats receiving TAM and CPA compared to those receiving only CPA. A total of 24 DELs were upregulated and 25 downregulated by tamoxifen. The identified DELs shared the characteristics of noncoding RNAs described in other reproductive tissues. Eleven of the identified DELs displayed divergent modes of action, regulating target transcripts via both cis- and trans-acting pathways. Functional enrichment analysis revealed that, among target genes ascribed to the identified DELs, the majority were involved in apoptosis, cell adhesion, immune response, and ovarian aging. The presented data suggest that the molecular mechanisms behind tamoxifen’s protective effects in the ovaries may involve lncRNA-dependent regulation of critical signaling pathways related to inhibition of follicular transition and ovarian aging, along with the suppression of apoptosis and regulation of cell adhesion. Employing a tumor-bearing animal model undergoing chemotherapy, which accurately reflects the conditions of mammary cancer, reinforces the obtained results. Given that tamoxifen remains a key player in the management and prevention of breast cancer, understanding its ovarian-specific actions in cancer patients is crucial and requires detailed functional studies to clarify the underlying molecular mechanisms.

## 1. Introduction

Thousands of premenopausal women are diagnosed with cancer annually and this number is increasing [1,2,3]. Advances in cancer treatment have not only extended lives but have also permitted cancer survivors to consider improving their quality of life. Standard cancer chemotherapy has long-lasting negative consequences for the female reproductive system [4,5]. Infertility due to ovarian toxicity is a major side effect of cancer treatment. Alkylating agents, such as cyclophosphamide (CPA), act by chemically interacting with DNA and pose a particular threat to the fertility of women who have survived cancer [6,7]. This interaction permanently damages gonadal tissue, including oocytes [8,9]. Well-known consequences of exposing female gonads to cytotoxic cancer therapies include a decrease in the oocyte reserve and the loss of ovarian function. Follicular loss predisposes women to amenorrhea, premature menopause, and infertility [10,11].

Improved cancer treatment and higher survival rates in cancer patients have allowed the development of the scientific and clinical discipline called oncofertility. The main goal of oncofertility is to preserve fertility in cancer patients [12,13,14], and to date embryonic cryopreservation is the most common approach. However, this technology is not widely used because it usually requires having a current partner and applying hormonal priming that can delay cancer treatment. The method is also technically and logistically demanding, invasive, and expensive. In addition, embryonic cryopreservation is infeasible in pre-pubertal females and fails to protect future ovarian function, leaving the patient at risk of early menopause [15]. Ovarian tissue cryopreservation may overcome some of these limitations; however, it is still an experimental technique [16,17].

Tamoxifen (ICI 46474) belongs to a class of selective estrogen receptor modulators (SERMs) and elicits estrogen agonist or antagonist responses in a tissue-specific manner. A number of studies have shown that TAM alleviates the ovarian side effects of cancer treatment [18,19,20,21]. TAM blocked CPA-induced follicular toxicity in a rat model [22,23]. Similar results were obtained in vitro, where TAM reduced CPA-induced follicle loss in neonatal rat ovaries [24]. Although these studies provided some insights concerning processes affected by TAM, the mechanism of TAM’s action in the ovaries has not been established. In our recent study [25], we examined the protective effects of TAM towards chemotherapy-induced toxicity and identified genes and proteins involved in the action of TAM in the ovary.

In the current study, we aimed to identify noncoding RNAs, specifically lncRNAs, involved in the protective role of TAM against CPA-induced toxicity in the rat ovary. It was reported that noncoding RNAs are abnormally expressed in breast cancer and correlate with the occurrence of breast cancer and resistance to chemotherapy [26,27,28]. In addition, lncRNA expression is often tissue- and cell-type-specific, making lncRNAs particularly attractive as diagnostic biomarkers, prognostic factors, and specific therapeutic targets [29,30]. Therefore, a better understanding of lncRNA expression in the ovaries of female cancer patients is essential for developing new treatment strategies, as well as new possibilities for fertility preservation after cancer. The current study is one of the first TAM fertility preservation studies performed on rats with concurrent mammary neoplasia. This approach assessed both the shielding effects of TAM on the ovary and the interaction of TAM with CPA treatment for mammary cancer.

## 2. Results

### 2.1. RNA Sequencing and Identification of lncRNA

**Figure 1:**
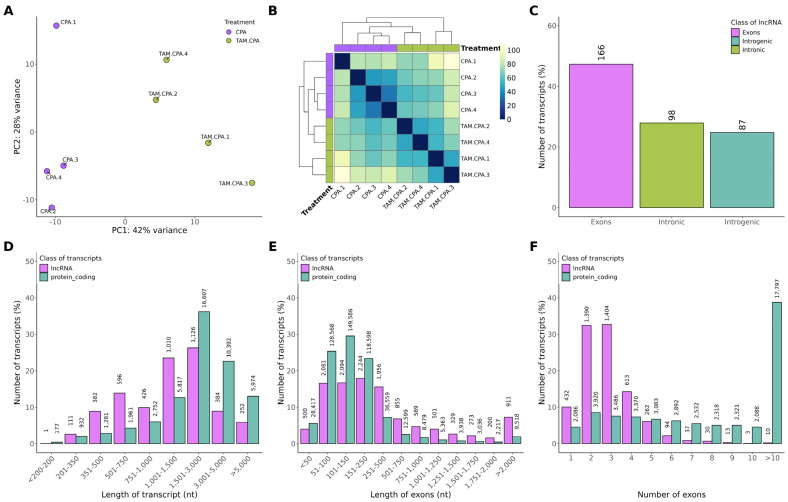
Characterization of the identified differentially expressed lncRNAs (DELs). (**A**) Graphical representation of the first (PC1) and second (PC2) principal components (PCs) affecting the lncRNA expression pattern, (**B**) distance matrix of differentially expressed lncRNAs (DELs; P-adjusted < 0.05 and log2FC ≥ |1.0|), and (**C**) genomic localization of the identified lncRNAs. (**D**–**F**)The comparison of genomic features (mean ± SEM) of the identified lncRNAs and mRNAs. The lncRNAs and mRNAs were compared in terms of average transcript length (**D**), exon length (**E**), and exon number (**F**). nt: nucleotides.

The sequencing data from the current study were submitted to the BioProject database under accession number PRJNA640997. Primary processing of raw reads yielded an average of 39,610,760 reads per sample (Table S1). The obtained reads were uniquely mapped to the rat reference genome (average of 35,458,204.38 mapped reads; average map efficiency exceeded 95%; Table S1) and assembled into transcripts (77,124 transcripts). Considering only the “x”, “o”, “i”, “u”, and “j” transcripts [31] allowed the selection of 16,898 transcripts for further analysis. Transcripts with lengths <200 bp and exon numbers < 2 (1992 transcripts) were removed due to potential bias. The random forest (RF) classifier final model, used to predict lncRNA, removed transcripts classified as mRNA from further analysis, leaving 359 transcripts predicted as potential lncRNA for further analysis. These transcripts were used to search orthologs using BLASTn. Transcripts were classified into three groups based on BLASTn results: (1) transcripts with a significant BLASTn match to a protein-coding gene (177) or non-lncRNA transcripts (16); (2) transcripts with a significant BLASTn match to a lncRNA (65); and (3) transcripts without a significant BLASTn match (101). The distance matrix and results of the Principal Component Analysis (PCA) revealed a high level of similarity between the biological replicates within each particular rat group (Figure 1A,B).

**Figure 2:**
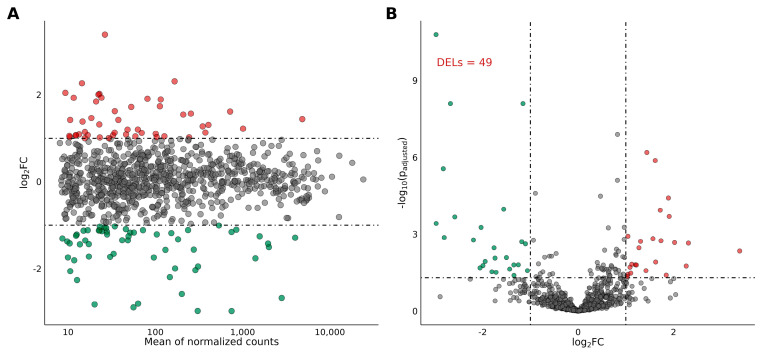
MA (**A**) and Volcano (**B**) plot presenting differentially expressed genes (DEGs; P-adjusted < 0.05 and log2FC ≥ |1.0|) and lncRNAs (DELs; P-adjusted < 0.05 and log2FC ≥ |1.0|) identified in the ovaries of tumor-bearing rats on the basis of the expression comparison performed between the CPA + TAM group and the CPA group. Red circles depict upregulated DEGs/DELs and green circles represent downregulated DEGs/DELs. Gray circles represent DEGs or DELs with no significant changes in expression.

The MA plot and Volcano plot present changes in the transcripts and lncRNA expression profiles of the ovaries collected from tumor-bearing rats treated with CPA + TAM in comparison to rats treated with CPA alone (Figure 2A,B).

### 2.2. Divergent Expression Patterns of lncRNA and mRNA

Transcript length, exon length, exon number, and expression level (mean ± SEM) were compared between all lncRNAs (known and novel) and mRNAs (Figure 1). According to statistical analysis of the lncRNA subtype, most transcripts were located within exons (Figure 1C). The average length of transcripts and exons, as well as the number of exons of lncRNA, was lower than that of mRNA (Figure 1D–F; Table S2). Transcripts of more than 1500 bp and less than 3000 bp predominated in both groups (Figure 1D). The majority of mRNAs (53.47%) had more than 10 exons, whereas the majority of lncRNAs (57.21%) consisted of two or three exons (Figure 1F).

### 2.3. Differentially Expressed lncRNAs and mRNAs in the Rat Ovary

In total, 166 lncRNAs were identified in the ovaries of rats, 49 of which were differentially expressed (P-adjusted < 0.05 and |log2FC|≥1.0) in the group receiving TAM together with CPA compared to the group receiving CPA alone (Table S3; Figure 2). Of the 49 identified DELs, 39 had been previously annotated in public databases. Among these, the expression of 21 DELs was upregulated by TAM, while the expression of 18 was downregulated. Ten of the identified DELs were classified as novel lncRNAs, with TAM increasing the expression of three and decreasing the expression of seven DELs. The log2FC value for DELs ranged from 3.38 (ENSRNOG00000069609) to −2.97 (ENSRNOG00000071212) (Table S3). The expression profiles of up- and downregulated DELs in the ovary of rats treated with CPA + TAM or CPA alone are presented in Figure 2. In addition, total of 23,096 mRNAs were identified in the ovaries, and 861 mRNAs of these were found to be differentially regulated (P adj. < 0.05 and log2FC ≥ |1.0|) in the group receiving TAM together with CPA in comparison to CPA group (Table S4).

### 2.4. The Cis- and Trans-Target Genes for DELs

**Figure 3:**
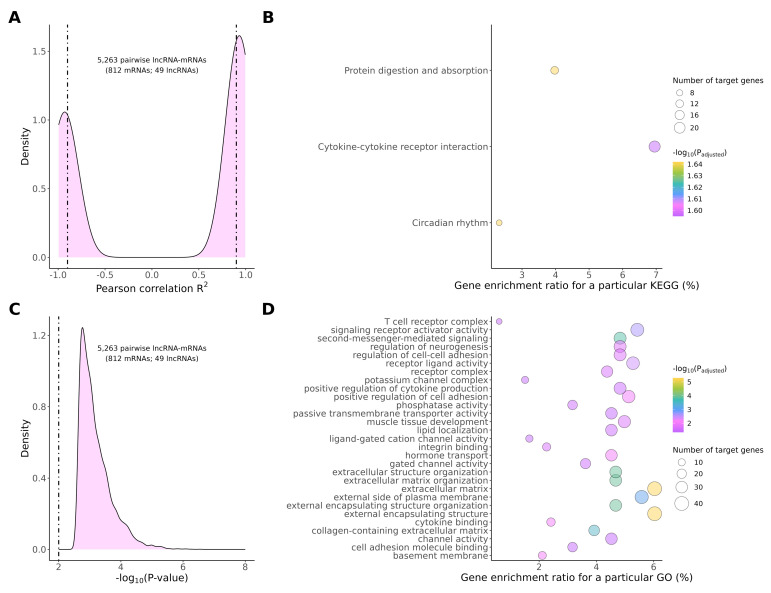
The visualization of the expression patterns of trans-regulated DEGs and DELs in the ovaries of tumor-bearing rats. (**A**) Pearson correlation between DELs and their potential DEG targets; (**B**) Kyoto Encyclopedia of Genes and Genomes (KEGG) analysis of target DEGs affected by DELs; (**C**) Statistical significance of the correlations presented in panel A; (**D**) Gene Ontology (GO) analysis of DEGs affected by DELs. The figure presents only GO subcategories containing the highest number of target DEGs.

To explore the regulatory mechanisms of trans-acting lncRNAs and their target mRNAs, co-expression analysis was performed based on DEGs and DELs using previously described methodology [32,33]. After filtration based on the *p*-value (*p* < 0.01) and Pearson’s correlation coefficient (|R2-value| > 0.9), 5263 pairwise lncRNA–mRNAs were identified (Figure 3A). These uniquely co-expressed transcript pairs included 812 DEGs and 49 DELs and the statistical significance of the correlation between co-expressed mRNA–lncRNA pairs was high (Figure 3A,C). To investigate the potential functional role of lncRNA, KEGG and GO enrichment analysis was applied using the co-expressed DEGs and DELs. The co-expressed DEGs were enriched in three main KEGG pathways—protein digestion and absorption, cytokine–cytokine receptor interactions, and circadian rhythm (Figure 3B; Table S5). Several significant GO terms were connected with extracellular matrix organization and remodeling, second-messenger-mediated signaling, signaling receptor activity, regulation of cell adhesion, and transmembrane activity (Figure 3D, Table S6).

The potential target DEGs for differentially expressed lncRNAs were used to explore the possible significance of these lncRNAs in molecular pathways underlying the protective role of TAM against CPA-induced toxicity in the rat ovary. In order to analyze the trans-type interactions between DELs and their target DEGs, co-expression analysis was performed. This analysis detected 5263 correlations, including 3169 negative and 2084 positive correlations (Table S7). All of the 49 identified DELs were found to potentially regulate multiple DEGs (Table S8). The target DEGs involved in lncRNA–mRNA correlations were enriched in 167 GO terms (biological process: 135, cellular components: eight, molecular function: 24), including second-messenger-mediated signaling (GO:0019932), regulation of cell–cell adhesion (GO:0022407), extracellular structure organization (GO:0043062), hormone transport (GO:0009914), the extracellular matrix (GO:0031012), and signaling receptor activator activity (GO:0030546) (Table S6).

**Figure 4:**
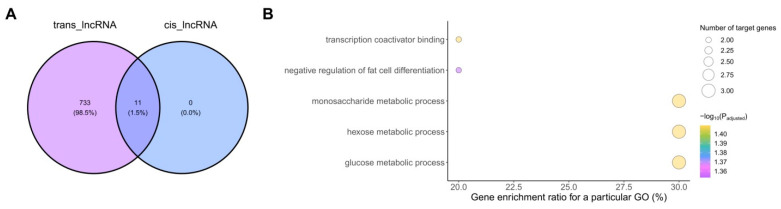
Identification (**A**) and functional enrichment analysis (**B**) of target DEGs potentially regulated by 11 DELs exhibiting divergent regulation of target gene expression (both in *cis-* and *trans-*).

Moreover, twenty-three cis-type correlations were identified, including five negative correlations and 18 positive correlations (Table S9). Of these, eleven DELs appeared to differently cis-regulate eleven DEGs (Table S10). The cis-regulated target DEGs were enriched (*p* < 0.05) in GO terms related to metabolic processes (GO:0019318, GO:0006006, GO:0005996), negative regulation of fat cell differentiation (GO:0045599), and transcription co-activator binding (GO:0001223; Figure 4B, Table S11). Additional analyses revealed that all eleven cis-regulating DELs were also interacting with distant transcripts in a trans-regulating manner (Figure 4A).

### 2.5. Validation of RNA-Seq Data by Real-Time PCR

**Figure 5:**
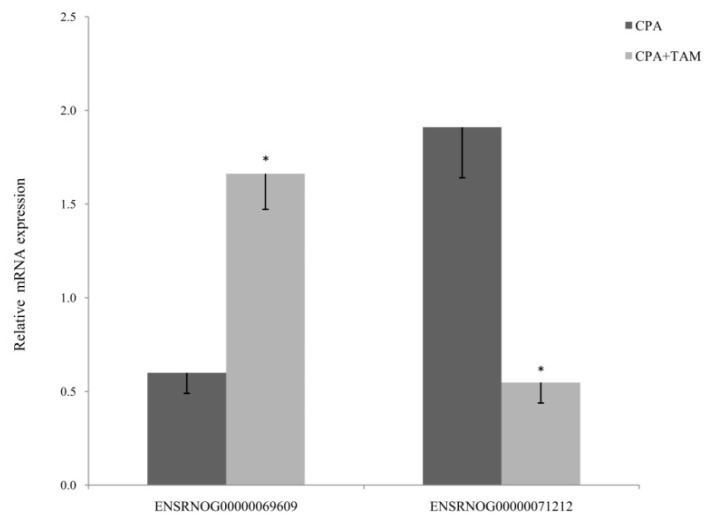
Real-Time PCR validation of the selected differentially expressed lncRNAs identified (RNA-Seq) in the ovaries of tumor-bearing rats (CPA + TAM vs. CPA). The asterisk depicts statistically significant differences (*p* < 0.05) in CPA+TAM group compared to CPA group.

Two differentially expressed lncRNAs obtained from RNA-Seq results were selected for real-time PCR (ENSRNOG00000069609 and ENSRNOG00000071212) to validate the RNA-Seq results. The expression of the selected DELs confirmed the results obtained by RNA-Seq (Figure 5).

## 3. Discussion

Recently, we have reported that tamoxifen administered together with CPA protected the ovary from chemotherapy-induced loss of ovarian follicles in rats without affecting cancer treatment [23]. The results supported our earlier reports [18,22,24], where the follicle number was analyzed in TAM-treated rats undergoing chemotherapy. It is important to emphasize that, in contrast to previous studies, the current experiment and that of Nynca et al. [23] were performed on the ovaries of rats bearing mammary tumors in a model of women with cancer undergoing chemotherapy. The potential mechanism of action of TAM was examined in the latter study with the use of transcriptomic and proteomic methods [23]. In the present study, we aimed to examine the regulatory potential of long noncoding RNAs in the protective action of TAM on these ovaries. We found that 49 lncRNAs were differentially expressed in the ovaries of tumor-bearing rats treated with CPA + TAM in comparison to CPA-treated rats. These differentially expressed lncRNAs (DELs) were ascribed to their potential target genes and the latter were enriched in 167 GO terms, reflecting their regulatory potential. The identified DELs were eventually analyzed in the context of their possible involvement in the regulation of genes that may be responsible for the protective effect of TAM on ovarian follicles. In recent years, several studies have identified an important role of lncRNAs in the regulation of ovarian follicle functions [34,35,36]. Differential expression of lncRNAs associated with reproductive processes was focused mostly on polycystic ovarian syndrome [37,38], endometriosis [39], and premature ovarian failure [40]. The lncRNAs identified in the current study shared their characteristics with lncRNAs demonstrated previously in reproductive tissues [37,38,39,40].

**Figure 6:**
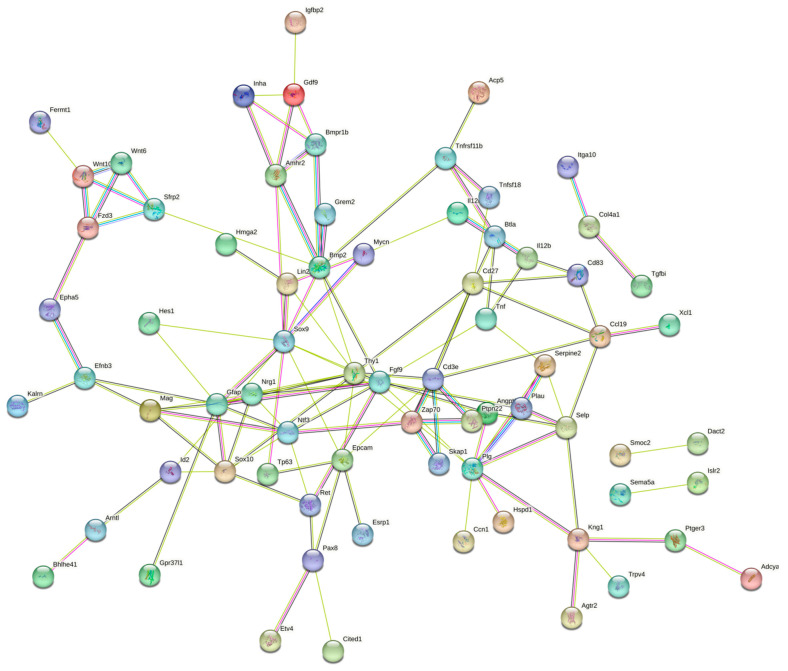
Interaction network of target DEGs potentially regulated by lncRNAs (DELs) identified in the ovaries of rats treated with cyclophosphamide (CPA) plus tamoxifen vs. rats treated with CPA alone. The network was generated by STRING (confidence score: 0.4) using DEGs (P-adjusted < 0.05 and log2 fold change ≥ 1.0) related to apoptosis and cell adhesion. Enrichment *p*-value: 1.0 × 10^−16^.

We have previously reported that the protective effect of TAM in the ovary of rats treated with CPA could result, at least partially, from decreased apoptosis in follicular cells [in vitro study: 24; in vivo study: 23]. Many DEGs identified in the ovarian transcriptome of TAM-treated rats with mammary tumors in the in vivo experiment [23] were associated with apoptosis. In the present study, 31 DELs were assigned to mRNA targets related to apoptosis (Tables S12 and S13). Some DELs were upregulated by TAM (Table S13), while others were downregulated (Table S12). In both cases, we identified apoptosis-associated DEGs (Figure 6), which were positively or negatively trans-correlated with DELs.

These data suggest that the majority of the apoptosis-associated DEGs may be regulated by numerous lncRNAs, and one lncRNA may affect the expression of many genes, indicating the complexity of processes that determine cell death or survival. Moreover, depending on the cell type or its physiological status, the proteins coded by the analyzed genes may occur in different isoforms and act on more than one type of receptor. Their effects may also depend on the activation or repression of other genes, as well as on the presence of other regulatory transcripts, e.g., miRNAs [41,42].

The ovarian expression of one of the identified DELs—ENSRNOG00000003984 (FC: −2.7)—was negatively correlated with the expression of the Adcyap1 gene, which encodes the pituitary adenylate cyclase-activating polypeptide (PACAP). Adcyap1 was found to be enriched in 30 GO terms, including those associated with gonadotropin response, hormone secretion, and intracellular signaling. Regarding the reproductive tract, the protein appears to be involved in the regulation of primordial germ cell proliferation and the cyclic recruitment of immature follicles, as well as steroid hormone and enzyme production in humans, rats, and cows [43]. In the current study, TAM administered with chemotherapy decreased the ovarian expression of ENSRNOG00000003984, leading to an increase in Adcyap1 expression. The possible association between TAM-induced increases in Adcyap1 expression (the present paper) and TAM-decreased apoptosis (our earlier papers [23,24]) in tumor-bearing rats treated with CPA is in agreement with previously published data concerning Adcyap1 involvement in the reduction of follicular apoptosis in rats [44]. The relationship between PACAP and the protective effect of TAM on ovarian follicles during cancer therapy needs to be investigated in detail.

Much evidence, including ovarian research, indicates a relationship between apoptosis and cell–cell contact, suggesting that apoptosis has an active role in reorganizing the cellular environment by inducing a rearrangement in cellular adhesion [45,46]. In the current study, bioinformatics analysis revealed that a number of differentially expressed lncRNAs were enriched in categories involved in the regulation of cell adhesion. A co-expression analysis of DELs and their predicted target genes indicated adhesion-associated molecules (e.g., epithelial cell adhesion molecule [EpCAM], Zap70, Skap1, plg, Plau, and Ptpn22), which may be involved in the lncRNA-mediated regulation of TAM’s protective actions in the ovary. Eight of these co-expressed DELs were related to the regulation of EpCAM. This type I transmembrane glycoprotein is expressed in normal epithelia [47,48], stem cells [49], and some cancers [50]. Zap70 (Zeta-chain-associated protein kinase 70), Skap1, or plg, in turn, influence cell adhesion and immune interactions [51,52,53,54]. The activity of EpCAM, zap70, or Skap1 is also related to cell proliferation, differentiation, and cellular signaling [47,50,55]. The bridging role of adhesion-related molecules—resulting from their pleiotropic actions—indicates these molecules as potential targets for targeted gene therapy in various types of cancers.

In our recent study, we reported that TAM can protect the ovary by inhibiting the transition of ovarian follicles. TAM upregulated the expression of inhibin α (Inha) and anti-Mullerian hormone (Amh), genes involved in signaling pathways related to primordial follicle activation or arrest [25]. These genes are known to be downregulated during the physiological primordial to primary follicle transition in rats [56], and TAM action seems to reverse the naturally occurring aging process. The Ovarian Aging Gene Signature (OAGS) represents the set of protein-coding genes and noncoding RNAs that constitute key elements of the transcriptomic profile of prematurely aging ovaries. The OAGS includes changes in the expression of genes such as Amh, Inha, Bmp, and Gdf9 [57,58,59] as well as those involved in lipid metabolism, lipid transport, and small molecule transport [60]. Similar to our previous study [25], we also found that TAM affected the expression of numerous DELs involved in the regulation of DEGs related to ovarian aging, including the above-mentioned Inha (14 DELs) and Amh (nine DELs). It is of interest that regardless of the mode of TAM action on the specific DELs (upregulation vs. downregulation) and the type of correlation between the DELs and their target DEGs (positive vs. negative correlation), TAM always increased the expression of DEGs (Inha and Amh). This finding supports our hypothesis that tamoxifen protects ovarian follicles by inhibiting their early transition. Moreover, in the current study, the ovarian expression of another DEL—ENSRNOG00000064307 (FC: 1.75)—was negatively correlated with the expression of the Ereg gene, which encodes epiregulin. In the reproductive system, epiregulin plays a role in stimulating the development of ovarian follicles and the maturation of oocytes [61,62]. In the present study, the ovarian expression of ENSRNOG00000064307 was significantly higher in the CPA + TAM group compared to animals receiving CPA, correlating with a decrease in Ereg gene expression. This further supports the notion that TAM may protect fertility during chemotherapy by inhibiting the transition and maturation of ovarian follicles.

The manner in which long noncoding RNAs regulate the expression of their target genes is pivotal for their biological function. It is known that cis-acting lncRNAs function locally, often being transcribed from the same genomic region as their target genes and performing their function by manipulating DNA or histone modifications [63]. In the current study, screening genes located within 20 kb from DELs enabled us to select 11 DELs acting in a cis manner. The target DEGs of these DELs were enriched in five GO terms involved in metabolic processes, fat cell differentiation, and regulation of transcription. It is suggested that cis-acting lncRNAs control their targets via spatial proximity determined by chromatin looping [64]. On the other hand, trans-acting lncRNAs function globally by regulating genes throughout the genome. To identify the trans-target genes presumptively regulated by lncRNAs, we correlated the expression level of DELs and protein-coding genes. The functional enrichment analysis of the co-expressed genes suggested a possible involvement of DELs in a variety of biological processes related to extracellular matrix rearrangement, apoptosis, cell adhesion, and signaling. Interestingly, the above-mentioned 11 DELs, at the same time, trans-regulated the expression of DEGs differently from those regulated in a *cis*-manner. A few examples previously showed that lncRNAs can function both near and distal to their transcribed locus [65,66]. However, how the cis and trans functions of lncRNAs are regulated and why some lncRNAs act in both capacities remain open questions.

The results of this study indicate that the molecular mechanisms underlying tamoxifen’s protective effects in the ovaries may be associated with the lncRNA-dependent regulation of key signaling pathways involved in the inhibition of follicular transition and ovarian aging, as well as the suppression of apoptosis and regulation of cell adhesion. The value of the results is enhanced by the usage of tumor-bearing animals undergoing chemotherapy as a model of mammary cancer. It must be emphasized that while direct extrapolation of these results to humans is not possible, our data highlight the need for further exploration of TAM’s impact on ovarian function in women undergoing oncological treatments. Functional studies employing knock-down or overexpressed lncRNAs to evaluate the significance of specific identified lncRNAs in the protective action of TAM in the ovaries are needed. Additionally, taking into account the fact that TAM continues to play an important role in both the management and prevention of breast cancer, understanding TAM’s ovarian-specific actions in cancer patients is of critical relevance and necessitates comprehensive mechanistic studies to elucidate the precise mechanisms at play.

## 4. Material and Methods

### 4.1. Animals and Treatments

**Figure 7:**
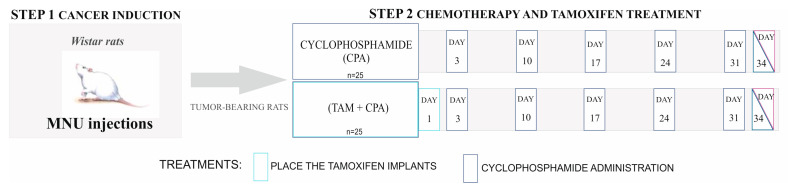
Experimental design of the study performed on rats. CPA was injected intraperitoneally, *n* = 25/group (modified from Nynca et al., 2023 [23]).

All procedures involving rats were approved by the Animal Ethics Committee of the University of Warmia and Mazury in Olsztyn, Poland (No. 78/2017/WNP). Female Wistar rats (6 weeks old, *n* = 125) were housed in a controlled environment (22 °C; 60% humidity; 12L:12D) in the Center of Experimental Medicine (Bialystok, Poland), with ad libitum access to food and water. To induce mammary gland tumors, *N*-methyl-*N*-nitrosourea (MNU; Toronto Research Chemicals, Canada; 50 mg/kg b.w.) was administered (ip) twice to 100 rats, at 7 and 19 weeks of age [11]. The remaining 25 rats, which constituted a non-tumor control group (CNT), received the vehicle (0.9% NaCl, 0.05% acetic acid) at these times. The presence of tumors or neoplastic lesions in rats was confirmed by a certified pathologist. A detailed description, as well as the weight of animals and tumor sizes, was reported in our previous paper [23]. At 31 weeks of age, the MNU-treated rats (*n* = 100), hereafter called tumor-bearing rats, were randomly assigned to the following groups (*n* = 25/group): 1/cyclophosphamide (CPA)-treated group and 2/(CPA + TAM)-treated group (Figure 7).

On day 1 of the experiment, CPA + TAM rats received subcutaneous implants that gradually released tamoxifen (1 mg/kg b.w./day; Innovative Research of America, Sarasota, USA). The dose of TAM was evaluated in terms of efficacy and toxicity in previous studies [18,19,22]. On day 3, CPA rats were injected (ip) with 50 mg/kg b.w. of CPA (Sigma, St. Louis, MO, USA; in 0.9% NaCl), followed by weekly injections (ip) of 10 mg/kg b.w. of CPA (days 10, 17, 24, and 31; Figure 7). The dose of CPA was carefully chosen on the basis of our previous experiments and available reports [15,22,23,24]. All rats were sacrificed on day 34 of the experiment; tissue samples were collected and the animals were checked for tumors. Ovaries were snap-frozen in liquid nitrogen and stored at −80 °C.

### 4.2. RNA Library Preparation and Sequencing

Total RNA was isolated from ovaries (*n* = 4 rats/group) using the peqGoldTriFast reagent. RNA concentration and quality were determined spectrophotometrically (NanoVue Plus, GE Healthcare, Little Chalfont, UK) and microfluidic electrophoresis (2100 Bioanalyzer; Agilent Technologies, Santa Clara, CA, USA) was employed to assess RNA purity and integrity. Samples with an RNA integrity number (28 S/18 S ratio) of at least 8.0 were used for RNA-Seq performed by Macrogen (Seoul, Republic of Korea). Total RNA was used to construct cDNA libraries (TruSeq stranded mRNA Sample Preparation Kit, Illumina, San Diego, CA, USA). A NovaSeq6000 high-throughput sequencing instrument (Illumina) was used for 100 bp paired-end configuration sequencing. A detailed sequencing methodology can be found in Nynca et al., 2023 [23].

### 4.3. Identification of lncRNA

The quality of raw reads was evaluated using the FASTQC tool (https://www.bioinformatics.babraham.ac.uk/projects/fastqc/, accessed on October 2022). Low-quality reads and adapters were removed using Trimmomatic software (version 0.39; [66]). The resulting reads were mapped to the rat reference genome (mRatBN7.2; Ensembl release 107) using STAR software (version 2.7.10a; [67]). Mapped reads were assembled into transcripts by StringTie (version 2.2.1; [68]). Assembled transcripts were then compared with reference annotation using Gffcompare (version 0.12.6; [31]). To predict novel lncRNAs, transcripts with class codes “x”, “o”, “i”, “u”, and “j” were retained [31]. Transcripts with length <200 nt and exon number < 2 were removed due to potential bias. The coding potentials of transcripts were evaluated using TransDecoder (version 5.5.0). Transcripts with an open reading frame (ORF) length < 300 nt (100 aa) were excluded from further analysis. Afterwards, the random forest (RF) classifier was used to create a mathematical model to identify lncRNAs. Briefly, nucleotide sequences of mRNA and lncRNAs for humans (GRCh38) and rats (mRatBN7.2) were downloaded from the Ensembl database (release 107). Transcripts with identical nucleotide sequences were deduplicated due to potential bias for both groups. LncFinder 1.1.4 was then used to extract features for each nucleotide sequence [69]. These features were used to create and train the RF classifier using 10-fold cross-validation by means of scikit-learn 1.1.2. The precision and recall of the RF model during hyperparameter tuning were evaluated on a test dataset. This RF classifier model was used to predict lncRNA, and transcripts classified as mRNA were removed from further analysis. Transcripts predicted as potential lncRNA were used to search orthologs using BLASTn (version 2.13.0; [70]) software against the NCBI Nucleotide Database (downloaded at 20 October 2022) with the E-value threshold set to 10-5. Transcripts were classified into three groups: (1) transcripts with a significant BLASTn match to a protein-coding gene or non-lncRNA transcripts; (2) transcripts with a significant BLASTn match to a lncRNA; and (3) transcripts without a significant BLASTn match. Transcripts classified to the first group were excluded from further analysis while transcripts from the second and third groups were used for future analysis as novel lncRNAs.

### 4.4. Identification of Differentially Expressed lncRNAs and Genes

After the identification of novel lncRNA, raw counts per gene were calculated using featureCounts (version 2.0.3; [71]). The differentially expressed lncRNAs (DELs) and genes (DEGs), as well as corresponding P-adjusted values, were determined by means of R statistical software (version 4.2.1) using the DESeq2 package (version 1.36.0; [72]). The threshold for significantly different expression levels was set at P-adjusted < 0.05 and log2 fold change (log2FC) ≥ 1.0 or log2FC ≤ −1.0. The visual presentation of the results was performed with R software using the ggplot2 (version 3.3.6; [73]) and pheatmap (version 1.0.12; [74]) packages.

### 4.5. Prediction of lncRNA Targets

The Pearson’s correlation coefficient between the expression profile of DEGs and DELs was determined using the Hmisc package (version 4.6-0) implemented into R statistical software (version 4.2.1). The threshold for trans-regulated DEGs was set as *p*-value < 0.05 and Pearson’s correlation coefficient R^2^ ≥ 0.90 or R^2^ ≤ −0.90. To identify cis-regulated genes, DEGs located within a distance of less than 20 kb from DELs were evaluated. Only pairs with *p*-value < 0.05 and R^2^ ≥ 0.90 or R^2^ ≤ −0.90 were considered as cis-regulated DEGs.

### 4.6. Functional Enrichment Analysis

Functional analysis of the identified targets for DEGs and DELs was performed based on the Gene Ontology (GO) database using the clusterProfiler (version 4.4.4; [75]), DOSE (version 3.22.1; [76]), biomaRt (version 2.52.0; [77]), and org.Rn.eg.db (version 3.15.0; [78]) packages of R software, with the established criterion P-adjusted ≤ 0.05. Additionally, the Kyoto Encyclopedia of Genes and Genomes (KEGG database) was used to ascribe identified lncRNA targets to particular biological mechanisms and cellular pathways (the established criteria: P-adjusted < 0.05). The KEGG enrichment analysis was performed using the clusterProfiler (version 4.4.4), DOSE (version 3.22.1), and org.Rn.eg.db (version 3.15.0) packages of R software. The visual presentation of the results was performed by R software using ggplot2 (version 3.3.6).

### 4.7. Validation of the Results by Real-Time PCR

Real-time PCR was used to validate the results of RNA-Seq by measuring the expression of two selected DELs identified in the ovaries of tumor-bearing rats treated with CPA + TAM vs.rats treated with CPA alone (ENSRNOG00000069609 and ENSRNOG00000071212). The validation was performed on the RNA samples used for RNA-Seq (*n* = 4 rats/group). Complementary DNA was reverse-transcribed from 1 µg of total RNA isolated from biological replicates using the Omniscript RT Kit (Qiagen, Hilden, Germany) with 0.5 μM oligo(dT)15 primer (Roche, Basel, Switzerland), 1 μM hexanucleotide primers, and 10 U RNase Out (SigmaAldrich, St. Louis, MO, USA) in a Veriti Thermal Cycler (Thermofisher Scientific, Waltham, MA, USA) at 37 °C for 1 h. Glyceraldehyde 3-phosphate dehydrogenase (GAPDH) and β-actin were used as reference genes. qRT-PCR was performed using the TaqMan Universal PCR Master Mix and TaqMan Gene Expression Assay (Thermofisher Scientific) in an Applied Biosystems7500 Fast Real-Time PCR System (Thermofisher Scientific). The amplification cycle was as follows: initial denaturation at 95 °C for 10 min, 40 cycles of denaturation at 95 °C for 15 s, and primer annealing at 60 °C for 1 min. qRT-PCR for each of four biological replicates was carried out in duplicate and a non-template control was included in each run. The gene expression level was normalized to GAPDH and β-actin to attain the relative expression using the comparative cycle threshold (CT) method and the quantity-based active schematic estimating (Q-BASE) model, and it was expressed as arbitrary units (mean ± SEM). The differences in lncRNA expression level between samples were evaluated using a Student’s t-test (Statistica Software Inc., Tulsa, OK, USA). Differences with a probability of *p*-value <0.05 were considered significant.

## Data Availability

All relevant data are within the paper and its Supporting Information files were deposited in the BioProject database under accession number PRJNA640997.

## Supplementary Materials

The following supporting information can be downloaded at: https://www.mdpi.com/article/10.3390/ijms252312538/s1.

## References

[1] Sung H., Ferlay J., Siegel R.L., Laversanne M., Soerjomataram I., Jemal A., Bray F. (2021). Global Cancer Statistics 2020: GLOBOCAN Estimates of Incidence and Mortality Worldwide for 36 Cancers in 185 Countries. CA A Cancer J Clin..

[2] Ugai T., Sasamoto N., Lee H.-Y., Ando M., Song M., Tamimi R.M., Kawachi I., Campbell P.T., Giovannucci E.L., Weiderpass E. (2022). Is Early-Onset Cancer an Emerging Global Epidemic? Current Evidence and Future Implications. Nat. Rev. Clin. Oncol..

[3] Zhao J., Xu L., Sun J., Song M., Wang L., Yuan S., Zhu Y., Wan Z., Larsson S., Tsilidis K. (2023). Global Trends in Incidence, Death, Burden and Risk Factors of Early-Onset Cancer from 1990 to 2019. BMJ Oncol..

[4] Meirow D., Nugent D. (2001). The Effects of Radiotherapy and Chemotherapy on Female Reproduction. Hum. Reprod. Update.

[5] Stroud J., Mutch D., Rader J., Powell M., Thaker P.H., Grisby P.W. (2009). Effects of cancer treatment on Ovarian Function. Fertil. Steril..

[6] Zhang H., Lu Y., Zhang Y., Dong L., Jiang S., Tang Y. (2024). DHA-enriched phosphatidylserine ameliorates cyclophosphamide-induced liver injury via regulating the gut-liver axis. Int. Immunopharmacol..

[7] Luan Y., Edmonds M.E., Woodruff T.K., Kim S. (2019). Inhibitors of apoptosis protect the ovarian reserve from cyclophosphamide. J. Endocrinol..

[8] Salama M., Winkler K., Murach K.F., Seeber B., Ziehr S.C., Wildt L. (2013). Female Fertility Loss and Preservation: Threats and Opportunities. Ann. Oncol..

[9] Hao X., Anastácio A., Liu K., Rodriguez-Wallberg K.A. (2019). Ovarian Follicle Depletion Induced by Chemotherapy and the Investigational Stages of Potential Fertility-Protective Treatments—A Review. Int. J. Mol. Sci..

[10] Spears N., Lopes F., Stefansdottir A., Rossi V., De Felici M., Anderson R.A., Klinger F.G. (2019). Ovarian Damage from Chemotherapy and Current Approaches to Its Protection. Hum. Reprod. Update.

[11] Kim S.S. (2006). Fertility Preservation in Female Cancer Patients: Current Developments and Future Directions. Fertil. Steril..

[12] Loren A.W., Mangu P.B., Beck L.N., Brennan L., Magdalinski A.J., Partridge A.H., Quinn G., Wallace W.H., Oktay K. (2013). Fertility Preservation for Patients With Cancer: American Society of Clinical Oncology Clinical Practice Guideline Update. JCO.

[13] Oktay K., Harvey B.E., Loren A.W. (2018). Fertility Preservation in Patients with Cancer: ASCO Clinical Practice Guideline Update Summary. JOP.

[14] Arecco L., Ruelle T., Martelli V., Boutros A., Latocca M.M., Spinaci S., Marrocco C., Massarotti C., Lambertini M. (2021). How to Protect Ovarian Function before and during Chemotherapy?. J. Clin. Med..

[15] Oktay K., Buyuk E., Davis O., Yermakova I., Veeck L., Rosenwaks Z. (2003). Fertility preservation in breast cancer patients: IVF and embryo cryopreservation after ovarian stimulation with tamoxifen. Hum. Reprod..

[16] Sauvat F., Bouilly J., Capito C., Lefèvre A., Blachère T., Borenstein N., Sarnacki S., Dandolo L., Binart N. (2013). Ovarian Function Is Restored after Grafting of Cryopreserved Immature Ovary in Ewes. FASEB J..

[17] Maltaris T., Beckmann M.W., Dittrich R. (2009). Fertility Preservation for Young Female Cancer Patients. In Vivo.

[18] Ting A.Y., Petroff B.K. (2015). Challenges and Potential for Ovarian Preservation with SERMs. Biol. Reprod..

[19] Kim S.S., Lee J.R., Jee B.C., Suh C.S., Kim S.H., Ting A., Petroff B. (2010). Use of Hormonal Protection for Chemotherapy-Induced Gonadotoxicity. Clin. Obstet. Gynecol..

[20] Mahajan N. (2015). Fertility preservation in female cancer patients: An overview. J. Hum. Reprod. Sci..

[21] Chuai Y., Xu X., Wang A. (2012). Preservation of Fertility in Females Treated for Cancer. Int. J. Biol. Sci..

[22] Ting A.Y., Petroff B.K. (2010). Tamoxifen Decreases Ovarian Follicular Loss from Experimental Toxicant DMBA and Chemotherapy Agents Cyclophosphamide and Doxorubicin in the Rat. J. Assist. Reprod. Genet..

[23] Nynca A., Swigonska S., Ruszkowska M., Sadowska A., Orlowska K., Molcan T., Myszczynski K., Otrocka-Domagala I., Paździor-Czapula K., Kurowicka B. (2023). Tamoxifen Decreases Ovarian Toxicity without Compromising Cancer Treatment in a Rat Model of Mammary Cancer. BMC Genom..

[24] Piasecka-Srader J., Blanco F.F., Delman D.H., Dixon D.A., Geiser J.L., Ciereszko R.E., Petroff B.K. (2015). Tamoxifen Prevents Apoptosis and Follicle Loss from Cyclophosphamide in Cultured Rat Ovaries. Biol. Reprod..

[25] Nynca A., Swigonska S., Molcan T., Petroff B.K., Ciereszko R.E. (2023). Molecular Action of Tamoxifen in the Ovaries of Rats with Mammary Neoplasia. Int. J. Mol. Sci..

[26] Majidinia M., Yousefi B. (2016). DNA Damage Response Regulation by MicroRNAs as a Therapeutic Target in Cancer. DNA Repair..

[27] Jin H., Du W., Huang W., Yan J., Tang Q., Chen Y., Zou Z. (2021). LncRNA and Breast Cancer: Progress from Identifying Mechanisms to Challenges and Opportunities of Clinical Treatment. Mol. Ther.-Nucleic Acids.

[28] Sideris N., Dama P., Bayraktar S., Stiff T., Castellano L. (2022). LncRNAs in Breast Cancer: A Link to Future Approaches. Cancer Gene Ther..

[29] Slack F.J., Chinnaiyan A.M. (2019). The Role of Non-Coding RNAs in Oncology. Cell.

[30] Nandwani A., Rathore S., Datta M. (2021). LncRNAs in Cancer: Regulatory and Therapeutic Implications. Cancer Lett..

[31] Pertea G., Pertea M. (2020). GFF Utilities: GffRead and GffCompare. F1000Res.

[32] Liu Y.R., Jiang Y.Z., Xu X.E., Hu X., Yu K.D., Shao Z.M. (2016). Comprehensive transcriptome profiling reveals multigene signatures in triple-negative breast cancer. Clin. Cancer. Res..

[33] Zhang S., Wang Y., Jia L., Wen X., Du Z., Wang C., Hao Y., Yu D., Zhou L., Chen N. (2019). Profiling the long noncoding RNA interaction network in the regulatory elements of target genes by chromatin in situ reverse transcription sequencing. Genome Res..

[34] Bouckenheimer J., Assou S., Riquier S., Hou C., Philippe N., Sansac C., Lavabre-Bertrand T., Commes T., Lemaître J.-M., Boureux A. (2016). Long Non-Coding RNAs in Human Early Embryonic Development and Their Potential in ART. Hum. Reprod. Update.

[35] Tu J., Chen Y., Li Z., Yang H., Chen H., Yu Z. (2020). Long Non-Coding RNAs in Ovarian Granulosa Cells. J. Ovarian Res..

[36] Usman M., Li A., Wu D., Qinyan Y., Yi L.X., He G., Lu H. (2024). The Functional Role of LncRNAs as CeRNAs in Both Ovarian Processes and Associated Diseases. Non-Coding RNA Res..

[37] Fu L., Xu Y., Li D., Dai X., Xu X., Zhang J., Ming H., Zhang X., Zhang G., Ma Y. (2018). Expression Profiles of mRNA and Long Noncoding RNA in the Ovaries of Letrozole-Induced Polycystic Ovary Syndrome Rat Model Through Deep Sequencing. Gene.

[38] Zhou W., Zhang T., Lian Y., Zhang W., Yang M., Li Y., Wang L., Yan X. (2022). Exosomal LncRNA and mRNA Profiles in Polycystic Ovary Syndrome: Bioinformatic Analysis Reveals Disease-Related Networks. Reprod. BioMedicine Online.

[39] Hudson Q.J., Proestling K., Perricos A., Kuessel L., Husslein H., Wenzl R., Yotova I. (2021). The Role of Long Non-Coding RNAs in Endometriosis. Int. J. Mol. Sci..

[40] Dong L., Xin X., Chang H.-M., Leung P.C.K., Yu C., Lian F., Wu H. (2022). Expression of Long Noncoding RNAs in the Ovarian Granulosa Cells of Women with Diminished Ovarian Reserve Using High-Throughput Sequencing. J. Ovarian Res..

[41] Ghafouri-Fard S., Hajiesmaeili M., Shoorei H., Bahroudi Z., Taheri M., Sharifi G. (2021). The Impact of LncRNAs and MiRNAs in Regulation of Function of Cancer Stem Cells and Progression of Cancer. Front. Cell Dev. Biol..

[42] Guerrache A., Micheau O. (2024). TNF-Related Apoptosis-Inducing Ligand: Non-Apoptotic Signalling. Cells.

[43] Koppan M., Nagy Z., Bosnyak I., Reglodi D. (2022). Female Reproductive Functions of the Neuropeptide PACAP. Front. Endocrinol..

[44] Lee J., Park H.-J., Choi H.-S., Kwon H.-B., Arimura A., Lee B.-J., Choi W.-S., Chun S.-Y. (1999). Gonadotropin Stimulation of Pituitary Adenylate Cyclase-Activating Polypeptide (PACAP) Messenger Ribonucleic Acid in the Rat Ovary and the Role of PACAP as a Follicle Survival Factor*. Endocrinology.

[45] Suzanne M., Steller H. (2009). Letting Go: Modification of Cell Adhesion during Apoptosis. J. Biol..

[46] Kawamoto Y., Nakajima Y., Kuranaga E. (2016). Apoptosis in Cellular Society: Communication between Apoptotic Cells and Their Neighbors. Int. J. Mol. Sci..

[47] Maetzel D., Denzel S., Mack B., Canis M., Went P., Benk M., Kieu C., Papior P., Baeuerle P.A., Munz M. (2009). Nuclear Signalling by Tumour-Associated Antigen EpCAM. Nat. Cell Biol..

[48] Van Der Gun B.T.F., Melchers L.J., Ruiters M.H.J., De Leij L.F.M.H., McLaughlin P.M.J., Rots M.G. (2010). EpCAM in Carcinogenesis: The Good, the Bad or the Ugly. Carcinogenesis.

[49] Tang D., Chen Y., Fu G.-B., Yuan T.-J., Huang W.-J., Wang Z.-Y., Li W.-J., Jiao Y.-F., Yu W.-F., Yan H.-X. (2020). EpCAM Inhibits Differentiation of Human Liver Progenitor Cells into Hepatocytes in Vitro by Activating Notch1 Signaling. Biochem. Biophys. Res. Commun..

[50] Xiao D., Xiong M., Wang X., Lyu M., Sun H., Cui Y., Chen C., Jiang Z., Sun F. (2024). Regulation of the Function and Expression of EpCAM. Biomedicines.

[51] Huber R.G., Fan H., Bond P.J. (2015). The Structural Basis for Activation and Inhibition of ZAP-70 Kinase Domain. PLoS Comput. Biol..

[52] Chen J., Moore A., Ringshausen I. (2020). ZAP-70 Shapes the Immune Microenvironment in B Cell Malignancies. Front. Oncol..

[53] Dadwal N., Mix C., Reinhold A., Witte A., Freund C., Schraven B., Kliche S. (2021). The Multiple Roles of the Cytosolic Adapter Proteins ADAP, SKAP1 and SKAP2 for TCR/CD3 -Mediated Signaling Events. Front. Immunol..

[54] Liu C., Raab M., Gui Y., Rudd C.E. (2023). Multi-Functional Adaptor SKAP1: Regulator of Integrin Activation, the Stop-Signal, and the Proliferation of T Cells. Front. Immunol..

[55] Cuomo D., Porreca I., Ceccarelli M., Threadgill D.W., Barrington W.T., Petriella A., D’Angelo F., Cobellis G., De Stefano F., D’Agostino M.N. (2018). Transcriptional Landscape of Mouse-Aged Ovaries Reveals a Unique Set of Non-Coding RNAs Associated with Physiological and Environmental Ovarian Dysfunctions. Cell Death Discov..

[56] Chen Y., Yang W., Shi X., Zhang C., Song G., Huang D. (2020). The Factors and Pathways Regulating the Activation of Mammalian Primordial Follicles in vivo. Front. Cell Dev. Biol..

[57] Cuomo D., Ambrosino C. (2019). Non-Coding RNAs as Integrators of the Effects of Age, Genes, and Environment on Ovarian Aging. Cell Death Dis..

[58] Ansere V.A., Ali-Mondal S., Sathiaseelan R., Garcia D.N., Isola J.V.V., Henseb J.D., Saccon T.D., Ocañas S.R., Tooley K.B., Stout M.B. (2021). Cellular Hallmarks of Aging Emerge in the Ovary Prior to Primordial Follicle Depletion. Mech. Ageing Dev..

[59] Chacón C., Mounieres C., Ampuero S., Urzúa U. (2023). Transcriptomic analysis of the aged nulliparous mouse ovary suggests a stress state that promotes pro-inflammatory lipid signaling and epithelial cell enrichment. Int. J. Mol. Sci..

[60] Riese D.J., Cullum R.L. (2014). Epiregulin: Roles in Normal Physiology and Cancer. Semin. Cell Dev. Biol..

[61] Richani D., Gilchrist R.B. (2018). The Epidermal Growth Factor Network: Role in Oocyte Growth, Maturation and Developmental Competence. Hum. Reprod. Update.

[62] Arunkumar G. (2024). LncRNAs: The Good, the Bad, and the Unknown. Biochem. Cell Biol..

[63] Gil N., Ulitsky I. (2018). Production of Spliced Long Noncoding RNAs Specifies Regions with Increased Enhancer Activity. Cell Syst..

[64] Luo S., Lu J.Y., Liu L., Yin Y., Chen C., Han X., Wu B., Xu R., Liu W., Yan P. (2016). Divergent LncRNAs Regulate Gene Expression and Lineage Differentiation in Pluripotent Cells. Cell Stem Cell.

[65] Stewart G.L., Sage A.P., Enfield K.S.S., Marshall E.A., Cohn D.E., Lam W.L. (2021). Deregulation of a Cis-Acting LncRNA in Non-Small Cell Lung Cancer May Control HMGA1 Expression. Front. Genet..

[66] Bolger A.M., Lohse M., Usadel B. (2014). Trimmomatic: A Flexible Trimmer for Illumina Sequence Data. Bioinformatics.

[67] Dobin A., Davis C.A., Schlesinger F., Drenkow J., Zaleski C., Jha S., Batut P., Chaisson M., Gingeras T.R. (2013). STAR: Ultrafast Universal RNA-Seq Aligner. Bioinformatics.

[68] Pertea M., Pertea G.M., Antonescu C.M., Chang T.-C., Mendell J.T., Salzberg S.L. (2015). StringTie Enables Improved Reconstruction of a Transcriptome from RNA-Seq Reads. Nat. Biotechnol..

[69] Han S., Liang Y., Ma Q., Xu Y., Zhang Y., Du W., Wang C., Li Y. (2019). LncFinder: An Integrated Platform for Long Non-Coding RNA Identification Utilizing Sequence Intrinsic Composition, Structural Information and Physicochemical Property. Brief. Bioinform..

[70] Camacho C., Coulouris G., Avagyan V., Ma N., Papadopoulos J., Bealer K., Madden T.L. (2009). BLAST+: Architecture and Applications. BMC Bioinform..

[71] Liao Y., Smyth G.K., Shi W. (2014). FeatureCounts: An Efficient General Purpose Program for Assigning Sequence Reads to Genomic Features. Bioinformatics.

[72] Love M.I., Huber W., Anders S. (2014). Moderated Estimation of Fold Change and Dispersion for RNA-Seq Data with DESeq2. Genome Biol..

[73] Wickham H. (2009). Ggplot2: Elegant Graphics for Data Analysis.

[74] Kolde R. Pheatmap: Pretty Heatmaps. 2010, 1.0.12. https://CRAN.R-project.org/package=pheatmap.

[75] Yu G., Wang L.-G., Han Y., He Q.-Y. (2012). ClusterProfiler: An R Package for Comparing Biological Themes Among Gene Clusters. OMICS A J. Integr. Biol..

[76] Yu G., Wang L.-G., Yan G.-R., He Q.-Y. (2015). DOSE: An R/Bioconductor Package for Disease Ontology Semantic and Enrichment Analysis. Bioinformatics.

[77] Durinck S., Spellman P.T., Birney E., Huber W. (2009). Mapping Identifiers for the Integration of Genomic Datasets with the R/Bioconductor Package BiomaRt. Nat. Protoc..

[78] Carlson M. (2017). Org.Rn.Eg.Db. https://bioconductor.org/packages/release/data/annotation/html/org.Rn.eg.db.html.

