# Supplementary material for: The Role of lncRNAs in the Protective Action of Tamoxifen on the Ovaries of Tumor-Bearing Rats Receiving Cyclophosphamide
Source: Int J Mol Sci. 2024 Nov 22;25(23):12538. doi: 10.3390/ijms252312538 (PMC11640806; doi:10.3390/ijms252312538)
Supplement: Supplementary file 1 [file ijms-25-12538-s001.zip › Table S12.pdf]

Table S12. Interaction of TAM-inhibited DELs with apoptosis-associated DEGs

TAM ↓ DELs					
Nr	Nr DEL*	DEL	FC TAM	(+) correlation between DELs z a DEGs	(-) correlation between DELs and DEGs
1	1	ENSRNOG00000003984	-2.67	Tnfrsf11a, Fgf9	Tnf, Adcyap1
2	11	ENSRNOG00000063553	-2.82	Tnfrsf11a	Wnt6, Mycn, TGFbi
3	14	ENSRNOG00000064728	-2.80	Tnfrsf11a, Fgf9	Tnf
4	17	ENSRNOG00000065786	-1.10	---	Mycn, Inha, Gdf9, Grem2, Nrg1, Apcdd1
5	20	ENSRNOG00000066884	-1.35	---	Bmp2, Tnfrsf11b, Nrg1, Apcdd1
6	24	ENSRNOG00000067743	-1.25	---	Tnf10, Bmpr1b, Bmp2, Tnfrsf11b, Gdf9, Nrg1, Apcdd1
7	27	ENSRNOG00000069051	-2.58	Tnfrsf11a, Fgf9	---
8	28	ENSRNOG00000069177	-1.18	Tnfrsf11a, Fgf9	---
9	34	ENSRNOG00000070418	-1.06	---	Bmp2, Mycn
10	39	ENSRNOG00000071212	-2.97	Tnfrsf22	Tnfsf10, Bmp2, Mycn, Wnt6, Inha, Gre Amhr2
11	42	MSTRG.11753	-1.16	Tnfrsf22	Tnfsf10, Bmp2, Mycn, Wnt10a, Wnt6,Grem2, Nrg1, Apcdd1
12	43	MSTRG.14509	-1.99	Tnfrsf22	Amhr2
13	44	MSTRG.15774	-1.43	Tnfrsf22	Bmp2, Mycn, Amhr2, Tnfrsf11b, Grem2, Nrg1, Apcdd1
14	45	MSTRG.16626	-2.06	Tnfrsf22	Mycn, Amhr2, Inha, Gdf9, Grem2, Tgfbi, Apcdd1
15	46	MSTRG.16732	-2.03	Tnfrsf22	Tnfsf10, Mycn, Amhr2, Inha, Grem2, T
16	49	MSTRG.9726	-1.76	---	Bmp2, Nrg1, Apcdd1

*DEL numbers from Table 3 (*List of target DEGs predicted to be potentially trans-regulated by DELs identified in the ovaries of mammary gland tumor-bearing rats undergoing chemotherapy (CPA) and treated with TAM (CPA vs. CPA+TAM*)
