# Supplementary material for: The Role of lncRNAs in the Protective Action of Tamoxifen on the Ovaries of Tumor-Bearing Rats Receiving Cyclophosphamide
Source: Int J Mol Sci. 2024 Nov 22;25(23):12538. doi: 10.3390/ijms252312538 (PMC11640806; doi:10.3390/ijms252312538)
Supplement: Supplementary file 1 [file ijms-25-12538-s001.zip › Table S13.pdf]

Table S13. Interaction of TAM-stimulated DELs with apoptosis-associated DEGs

TAM ↑DELs					
Nr	**Nr DEL***	**DEL**	**FC TAM**	**(+) correlation between DELs and DEGs**	**(-) correlation between DELs and DEGs**
1	4	ENSRNOG00000062597	1.63	---	Tnfsf10, Bmp2, Mycn, Wnt10a, Wnt6, Inha, Gdf9, Grem2, Apcdd1
2	5	ENSRNOG00000062687	2.27	Tnfrsf22	Tnfsf10, Bmp2
3	6	ENSRNOG00000062688	2.02	---	Mycn, Wnt6,
4	9	ENSRNOG00000063238	1.09	---	Tnfsf10, Mycn, Wnt10, Wnt6, Inha, Gdf9, Grem2, Apcdd1
5	10	ENSRNOG00000063513	1.27	---	Tnfsf10, Bmp2, Mycn, Tnfrsf11b, Inha, Gdf9, Grem2, Apcdd1
6	12	ENSRNOG00000064307	1.74	Tnsf18	Tnfsf10, Bmp2, Mycn,Wnt6, Inha, Gdf9, Grem2, Apcdd1
7	15	ENSRNOG00000064809	1.72	---	Bmp2, Mycn, Apcdd1
8	19	ENSRNOG00000066588	1.03	---	Amh, Tnfrsf11b, Gdf9, Grem2,
9	21	ENSRNOG00000067151	1.62	---	Bmp2, Mycn, Wnt10a, Wnt6, Inha, Apcdd
10	22	ENSRNOG00000067312	1.2	Tnfrsf22	Mycn, Amh, Amhr2, Grem2, Apcdd1
11	29	ENSRNOG00000069588	1.57	Tnsf18	Tnfsf10, Mycn, Wnt10a, Wnt6, Inha
12	33	ENSRNOG00000070241	1.44	---	Bmp2, Mycn, Amhr2, Wnt10a, Wnt6, Inha, Gdf9, Grem2, Nrg1, Apcdd1
13	40	MSTRG.11066	1.42	---	Inha, Gdf9, Grem2, Nrg1, Apcdd1
14	41	MSTRG.11277	1.31	---	Amh, Tnfrsf11b, Grem2, Nrg1, Apcdd1
15	47	MSTRG.2163	1.89	Tnfrsf22	Tnfsf10, Bmp2, Wnt10a, Wnt6, Inha, Grem2,

*DEL numbers from Table 3 (*List of target DEGs predicted to be potentially trans-regulated by DELs identified in the ovaries of mammary gland tumor-bearing rats undergoing chemotherapy (CPA) and treated with TAM (CPA vs. CPA+TAM*)
